# A Colour Opponent Model That Explains Tsetse Fly Attraction to Visual Baits and Can Be Used to Investigate More Efficacious Bait Materials

**DOI:** 10.1371/journal.pntd.0003360

**Published:** 2014-12-04

**Authors:** Roger D. Santer

**Affiliations:** Institute of Biological, Environmental, and Rural Sciences, Aberystwyth University, Aberystwyth, Ceredigion, United Kingdom; International Centre of Insect Physiology and Ecology, Kenya

## Abstract

Palpalis group tsetse flies are the major vectors of human African trypanosomiasis, and visually-attractive targets and traps are important tools for their control. Considerable efforts are underway to optimise these visual baits, and one factor that has been investigated is coloration. Analyses of the link between visual bait coloration and tsetse fly catches have used methods which poorly replicate sensory processing in the fly visual system, but doing so would allow the visual information driving tsetse attraction to these baits to be more fully understood, and the reflectance spectra of candidate visual baits to be more completely analysed. Following methods well established for other species, I reanalyse the numbers of tsetse flies caught at visual baits based upon the calculated photoreceptor excitations elicited by those baits. I do this for large sets of previously published data for *Glossina fuscipes fuscipes* (Lindh et al. (2012). PLoS Negl Trop Dis 6: e1661), *G. palpalis palpalis* (Green (1988). Bull Ent Res 78: 591), and *G. pallidipes* (Green and Flint (1986). Bull Ent Res 76: 409). Tsetse attraction to visual baits in these studies can be explained by a colour opponent mechanism to which the UV-blue photoreceptor R7y contributes positively, and both the green-yellow photoreceptor R8y, and the low-wavelength UV photoreceptor R7p, contribute negatively. A tool for calculating fly photoreceptor excitations is made available with this paper, and this will facilitate a complete and biologically authentic description of visual bait reflectance spectra that can be employed in the search for more efficacious visual baits, or the analysis of future studies of tsetse fly attraction.

## Author Summary

Tsetse flies transmit sleeping sickness (human African trypanosomiasis), and visually attractive targets and traps are important tools for the control of the flies and prevention of disease. Previous studies have tried to determine the best colour for visual baits by relating their light reflectance properties to their attractiveness to tsetse. However, these methods represent only part of the visual information captured by the fly's eye, which is encoded by five different types of photoreceptor with varying sensitivities to different wavelengths of light. I use established methods to calculate the excitation of each fly photoreceptor type by the visual baits used to catch tsetse flies in three previous field studies. This method more completely describes the visual information captured by the fly's eye. Tsetse fly attraction can then be largely explained by a comparison of the excitations of three different photoreceptor types within the fly's nervous system. This knowledge and approach will allow for the more complete quantification of visual bait reflectance spectra, so that more efficient bait materials can be identified and employed to control tsetse flies.

## Introduction

Tsetse flies (*Glossina* spp.) are the vectors of trypanosomes that cause nagana in cattle, and sleeping sickness (human African trypanosomiasis, HAT) in humans [1]. There are no vaccines against HAT, no prophylactic drugs are recommended, and diagnosis and treatment of the disease is difficult [2], [3]. Control of tsetse flies is, therefore, of great importance for public health in sub-Saharan Africa [2], [3]. HAT is chiefly transmitted by riverine tsetse flies ( =  Palpalis species group) [3], and insecticide-treated screens and targets (two-dimensional cloth panels), and traps (three-dimensional structures) are an important part of control, eradication, and monitoring operations for these species [4], [5], [6]. The most effective visual bait material for catching tsetse flies is widely accepted to be phthalogen blue cotton (e.g. [5], [7]), but this material is now reportedly difficult to obtain, and modern synthetic fabrics are expected to be more durable and cost-effective for field use [5]. For all of these reasons, there has been considerable interest in understanding the attractive properties of visual baits so that they may be further optimised in terms of cost and efficacy [3], [4], [5], [8], [9].

Tsetse flies are caught at visual baits as a result of two behavioural processes: their initial attraction to approach the bait from a distance, and their tendency to land upon it (or enter it, in the case of a trap) once within range [10]. Several studies have attempted to relate the effectiveness of variously coloured visual baits at attracting tsetse flies or eliciting landing responses, to their reflectance at particular wavelengths of light, both for Palpalis group and Morsitans group (savannah) tsetse flies [5], [7], [11]. Sometimes these studies have considered total bait reflectance within several mutually exclusive wavelength bands [5], [7], [11], or more recently, point reflectance at the sensitivity peaks known for fly photoreceptors [5]. These analyses have indicated positive contributions of blue wavelengths, and negative contributions of green/yellow/red and UV wavelengths, to a visual bait's effectiveness at attracting tsetse [5], [7], [11]. In many investigations, the proportion of attracted flies that contacted the visual bait was positively influenced by its reflectance of UV wavelengths, or low overall luminance [7], [10], [12], [13] (but see also [5]). However, since only a relatively small proportion of the flies attracted to a bait actually land [5], [8], [10], [14], flanking nets of fine, insecticide-treated mesh are advocated to improve the efficiency of screens and targets by capturing non-landing, circling flies through accidental collisions with the net [8], [15].

**Figure 1:**
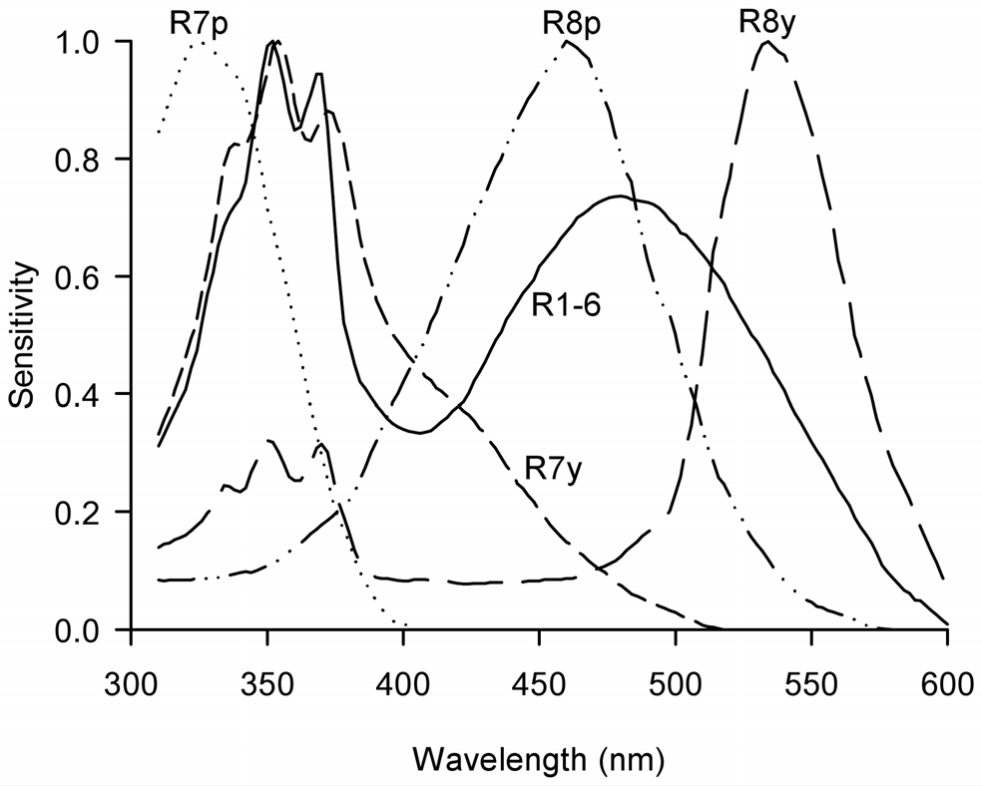
Normalised fly photoreceptor sensitivity functions as described by Hardie (1986) [17]. Each ommatidium of the compound eye contains eight photoreceptors, or retinula cells, R1-8. R1-6 are a homogenous group, found in every ommatidium of the eye. Over the majority of the eye, R7 and R8 occur in two subtypes, R7y and R8y in about 70% of ommatidia, and R7p and R8p in the remaining 30% [17]. Most fly photoreceptors have complex sensitivity functions that are not well described by their wavelength at peak sensitivity. This plot was produced by the author to show the photoreceptor sensitivity functions used in his analysis; the data underlying these were obtained from [17].

As a result of these investigations, it has been suggested that the search for new visual bait materials must be guided by full spectral analysis of them, rather than just visual inspection of candidate fabrics and qualitative description of their colour [5]. However, whilst the approaches used so far for such analyses have been motivated by the mechanisms of fly vision, they represent this process relatively crudely. Flies possess five classes of photoreceptor over the majority of a compound eye, and these have complicated sensitivity functions that both overlap with one another, and are not well-described by the single sensitivity peak of each receptor type [16], [17], [18] (see Fig. 1). The responses of these receptors do not just depend on the reflectance of a visual bait but also the predominant background that the bait is viewed against, because photoreceptors adapt to constant stimulation (e.g. [19]). Finally, the light reflected by a visual bait depends not only on its reflectance spectrum, but also the spectrum of light that it is illuminated with (e.g. [20]). Although the validity of findings relating visual bait effectiveness to reflectivity is certainly not in question, an improved, biologically-motivated method of analysing and quantifying the appearance of baits from the fly's eye view is clearly required for their further optimisation.

Methods to model photoreceptor responses taking into account the above described aspects of their response properties are now well established and have been widely employed to understand visually-guided behaviour in a variety of species (e.g. [21], [22], [23], [24], [25], [26]). Here, I apply these techniques to understand attraction to visual baits in three species of tsetse fly (two riverine and one savannah species) for which large datasets of tsetse catches at coloured visual baits were available in published studies [5], [7], [11]. Rather than attempting to analyse the positions of these visual baits within a fly colour space determined by the responses of all photoreceptor types (c.f. [22], [23], [27]), I employ regression methods to determine the subset of photoreceptors, and linear interactions between them, that best explain the behaviour of tsetse flies. A broadly similar approach has successfully identified opponent colour coding mechanisms in hymenoptera [28], and explained innate colour preferences in butterflies and flies seeking oviposition or feeding sites [24], [29]. This approach was chosen because the latter studies of innate colour preferences have revealed that the behaviours are often driven by only a subset of the photoreceptor types possessed by the subject organism [24], [29], [30]. On the basis of my analysis, I present a simple colour opponent model that explains attraction in all three tsetse fly species based upon photoreceptor excitations, and I make this available as electronic supporting material. This model provides a means to analyse visual bait reflectance spectra completely, and in a biologically-authentic manner, and can be employed for the analysis of candidate visual baits or of future studies of tsetse fly attraction.

## Methods

### Fly photoreceptor excitation model

Photoreceptor spectral sensitivity functions have been thoroughly characterised in *Musca* spp., and are similar across flies from other genera (e.g. [17]). Although photoreceptor spectral sensitivity functions have been recorded for *Glossina* spp., these were affected by a diet-induced lack of screening pigment that may or may not affect wild populations [18]. Since the overall organisation of tsetse photoreceptors was, nevertheless, similar to that in *Musca* [16], [17], [18], data from the latter were used in this study.

Each ommatidium in the dipteran compound eye contains eight photoreceptors, or retinula cells, named R1-R8. R1-6 are similar across all ommatidia in the eye, have a double-peaked spectral sensitivity function that peaks in UV and blue wavelengths, and make output synapses in the first neuropile of the optic lobe, the lamina [17]. Photoreceptors R7 and R8 are located centrally within each ommatidium and bypass the lamina to make output synapses in the medulla of the optic lobe. R7 and R8 occur in two forms across the majority of the eye (excluding specialised areas for perception of polarised light in both sexes, and for tracking of females in male flies) [17]. In 70% of these ommatidia the ‘y’ (yellow) form occurs in which R8y is most sensitive to green-yellow wavelengths but has an accessory, sensitising pigment sensitive to UV [16], [17], and R7y is most sensitive to UV wavelengths (∼355nm) with a pronounced shoulder of sensitivity extending into the blue region of the spectrum [16], [17]. In the remaining 30% of ommatidia, the ‘p’ (pale) form of these photoreceptors occurs, where R8p is most sensitive to blue wavelengths, and R7p to lower UV wavelengths (∼330nm) [16], [17]. Despite some anatomical and physiological differences between the R1-6 and R7/8 receptors, several mechanisms ensure that their responses are comparable [31]. For example, due to their structure and position within the ommatidium, photoreceptors R7 and R8 intercept less photons than R1-6, but they compensate for this with a greater voltage gain per photon so that overall, voltage gain per unit contrast is equalised across photoreceptor types [31]. As such, I employed the same method to model the responses of each photoreceptor type. Spectral sensitivity functions for each of the five receptor classes were extracted from published studies [16], [17] using DataThief software [32], and are plotted in Fig. 1. To avoid extrapolation beyond published data, spectral sensitivity functions were considered between 310 and 600nm, but the sensitivity of a fly's eye to wavelengths outside of this range is expected to be negligible.

In addition to their spectral sensitivity functions, the relative sensitivities of each receptor class are also determined by their adaptation to stimulation from the background. Following established methods that are accessibly described by Chittka and Kevan (2005) [26], the range sensitivity factor (R) was calculated for each receptor class in order to adjust their sensitivities such that background stimulation would elicit a half maximal response in each receptor class:
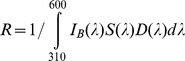



Where I_B_(λ) is the spectral reflectance function of the background; S(λ) is the spectral sensitivity function of a particular receptor (see Fig. 1); D(λ) is the illuminant; and dλ signifies a wavelength step for each of these functions. Following previous work, I used a standard illuminant function (D65, [33]) expressed as normalised quanta (values provided in [26]). Values for this function were available at 5 nm increments, and linear interpolation was used to achieve 2 nm wavelength resolution. I used published values for typical leaf reflectance as a background spectrum (values as provided in [26], linearly interpolated for a 2 nm wavelength resolution), which was a reasonable simplifying assumption given the typical riverine forest and thicket habitat of Palpalis group tsetse (e.g. figure 2 of [34]; figure S6 of [6]).

Based on these data, the effective quantum catch (P) of reflected light from a given visual bait, adjusted by R to reflect adaptation to the background, was calculated for each of a fly's five photoreceptor classes by:
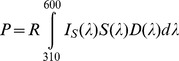



Where I_S_(λ) is the spectral reflectance function for the stimulus under investigation. Quantum catches were non-linearised to represent the transduction process in each photoreceptor, providing excitation (E) based upon:
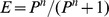



This equation has been used to describe intensity-response functions of photoreceptors across a variety of taxa. The exponent, n, determines the slope of the function and varies with the state of light adaption, approaching 1.0 in fully light-adapted photoreceptors (as demonstrated for R1-6 of flies, and also for locust photoreceptors [35]). As such, n = 1.0 was used in this study, and the above equation reduced to a simplified form without exponent (c.f. [26], [28]).

Using these methods, the relative excitation in each of the five photoreceptor classes could be calculated, accounting for the spectra of illumination and bait reflectance, the complete spectral sensitivity function of each photoreceptor, and adaptation of that photoreceptor to stimulation from the predominant background. A spreadsheet that performs these calculations is provided (Table S1), as is the complete dataset of photoreceptor excitations calculated during this study (S1 Dataset).

### Tsetse fly data

I applied the above analysis techniques to the datasets collected in the three most comprehensive studies of tsetse fly catches at coloured baits under field conditions conducted to date [5], [7], [11]. My focus was the initial attraction of tsetse flies to those baits (rather than their landing responses), since recent work optimising visual baits for Palpalis group tsetse flies recommends small targets with flanking nets, which are effective against both landing and circling flies [8]. My intention was not to meta-analyse colour attraction in tsetse, which has been investigated in multiple species, locations, and using a plethora of visual bait designs, all of which mean that the studies are not necessarily comparable. The three selected studies were identified through searches of Web of Science (Thomson Reuters), and of the cited references from studies identified in those searches, and from key review papers [10], [36]. The criteria for their selection were that (i) they each investigated a large sample (>25 in these cases) of visual baits that varied only in colour, and (ii) they provided full reflectance spectra from 300 to 700 nm for each visual bait. Thus, although numerous other studies have compared tsetse fly catches at visual baits that vary in colour, they were not analysed due to the lack of complete reflectance spectra (e.g. [37]), or the relatively small selection of colours investigated [12], [38], [39], [40].

Data on catches of *G. f. fuscipes* (Palpalis group) at 37 differently coloured small (0.25 m^2^) cloth targets were obtained from Lindh et al. (2012) [5]. This study was conducted on the Chamaunga islands of Lake Victoria, Kenya. Data were mean combined tsetse fly catches over both a surface electrocuting net (to sample flies landing on the cloth target), and an equal-sized flanking electrocuting net enclosing a fine, black mesh panel (to sample circling flies). Data were gained from 15 experiments in which combinations of five differently coloured targets were tested. A phthalogen blue standard target was included in each experiment, and mean tsetse fly catches for each target were normalised to that for the standard target [5]. These data were read directly from tables in the source publication. Lindh et al. (2012) provide reflectance spectra for each of their targets at 10 nm resolution, which I linearly interpolated to achieve 2 nm resolution for the purposes of analysis.

Data on catches of *G. p. palpalis* (Palpalis group) at large (1.0 m^2^) cloth screens in one experiment, and biconical traps in another, were obtained from Green (1988) [7]. This study was conducted in the Bouaflé area of Ivory Coast. Although cloth screen experiments examined a variety of electrocuting net configurations, I analysed only the data from experiments in which a surface net and one flanking net (1.0 m×0.5 m) were used (for which most data were available, and both landing and circling flies were sampled; data in tables IIIb, IV, and V, from [7]). Data were thus mean combined surface and flanking net tsetse fly catches for 27 screens, gained from 10 experiments in which four different screens were compared. As above, mean tsetse fly catches were normalised to that of a standard phthalogen blue screen in each experiment, although the reflectance spectrum for this stimulus was not exactly equivalent to that for the standard target of Lindh et al. (2012). Biconical trap experiment data were mean tsetse fly catches for 26 biconical traps with differently coloured lower cones, normalised to those of a biconical trap with standard phthalogen blue lower cone [7]. All biconical traps had standard black interior screens and an upper cone of white mosquito netting. Catch data were read directly from data tables, and screen/trap reflectance spectra were extracted from figures using DataThief software (measurements provided in supporting dataset S1).

I also analysed data on the catches of *G. pallidipes* (Morsitans group) in F2 traps from Green and Flint (1986) [11]. This study was conducted in the Zambezi valley, Zimbabwe. Data were mean tsetse fly catches for 30 F2 traps with differently coloured outer cloth covers but standard black interiors, gained from five experiments in which five or six differently coloured traps were tested. Trap catches were normalised by the catch of a white standard trap in each experiment. Reflectance spectra and normalised trap catch data were both extracted from figures using DataThief software (measurements provided in supporting dataset S1).

Since the above described target and screen experiments used surface and flanking electrocuting nets to sample both landing and circling flies, they provide a good indication of attraction to the visual baits that should be relatively little affected by any variation in landing responses across baits. The biconical and F2 trap experiments, meanwhile, sampled only those flies that entered the trap and were caught. These were expected to provide a less accurate indication of attraction since outer trap surfaces that have strong positive or negative influences on landing responses may have affected trap catches. For example, dark outer trap surfaces are believed to stimulate flies to land on them, rather than entering the trap and being sampled [10], [11].

### Statistical analysis

The original field studies asserted a log-log linear relationship between visual bait reflectance and normalised tsetse fly catches (e.g. [5], [11]), and on the basis of the same data I observed that calculated photoreceptor excitations related approximately linearly to log transformed normalised tsetse fly catches. Thus, for each study, tsetse fly catches were expressed as a percentage of the standard bait's catch, and log (n+1) transformed for analysis.

All available data are presented graphically, but statistical analyses were conducted on a sub-set of data in which each bait was represented once, against its mean normalised tsetse fly catch if presented multiple times within a given study (the latter collated dataset is provided; supporting dataset S1). Photoreceptor excitations were generally not normally distributed, so correlations between the excitation of different photoreceptors were assessed using Spearman's rank correlation. Linear regression was used to relate log transformed normalised tsetse fly catches to various combinations of calculated photoreceptor excitations, or indices derived from them. Due to highly multicollinear photoreceptor excitations, exploratory multiple regressions were carried out using the partial least squares (PLS) regression procedure. This procedure maps photoreceptor excitations and catch scores to a series of latent factors that explain their variability, and the number of latent factors was chosen so as to minimise predicted error sum of squares (PRESS), and maximise predicted r^2^. In one case it was felt that the model specified in this way was over-fitted due to a marginal improvement in these statistics versus a model with one latent factor less, and the simpler model was, therefore, selected on parsimony grounds (this case is identified in the relevant results table). Directed, follow-up linear regressions were then employed to test the models implicated by PLS regression statistically, through sequential addition of predictors with F tests of r^2^ change. PLS regression analyses were conducted using Minitab 14.20 (Minitab Inc., State College PA, USA); all other statistical analyses were conducted using SPSS version 19.0 (IBM Corp., Armonk NY, USA).

## Results

### Calculated photoreceptor excitations

**Figure 2:**
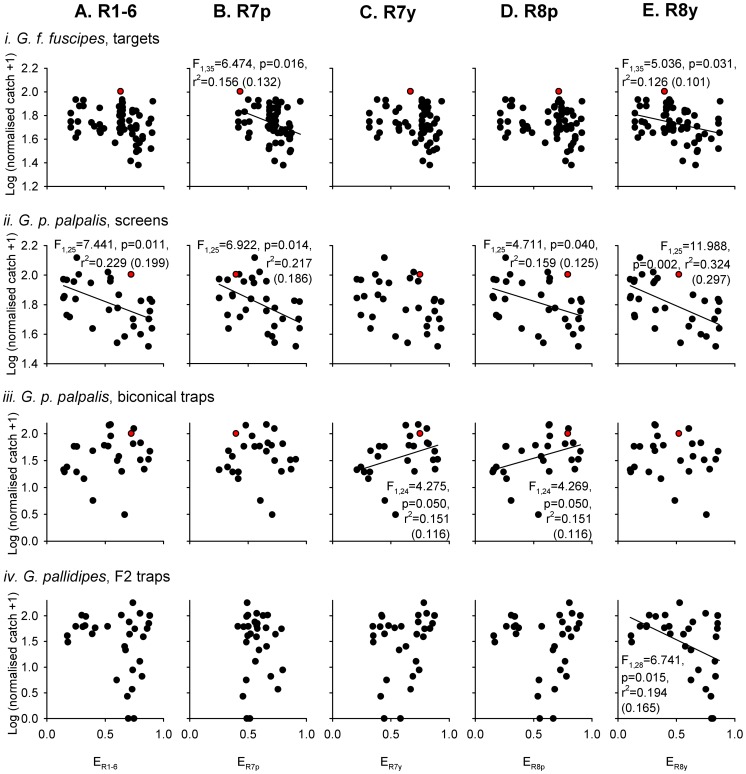
Male tsetse fly catches, and photoreceptor excitations, elicited by a range of visual baits. Male tsetse fly catches were obtained from three published field studies conducted on *G. f. fuscipes* using small targets with surface and flanking electrocuting nets (i) [5], *G. p. palpalis* using large screens with surface and flanking electrocuting nets (ii), or using biconical traps (iii) [7], and *G. pallidipes* using F2 traps (iv) [11]. Tsetse fly catches are expressed as a percentage of the catch of a standard, phthalogen blue (i)–(iii), or white (iv) equivalent, and have been log(n+1) transformed. Data for the phthalogen blue bait itself are indicated by the red data point in (i–iii). Tsetse catches are plotted against calculated excitations for each of the five main types of fly photoreceptor (A–E; refer to Fig. 1), based upon the reflectance spectra of visual baits used in the original studies. In each plot, data points are tsetse catches for every visual bait presentation in the original study, but linear regression analyses were conducted on a reduced dataset in which each visual bait was represented once (against its mean normalised tsetse fly catch): (i) 75 catch measurements, 37 different targets; (ii) 40 catch measurements, 27 different screens; (iii) 26 catch measurements, 26 different biconical traps; (iv) 33 catch measurements, 30 different F2 traps. The outcomes of statistical analyses are indicated where a significant relationship was identified (with adjusted r^2^ value in brackets).

**Figure 3:**
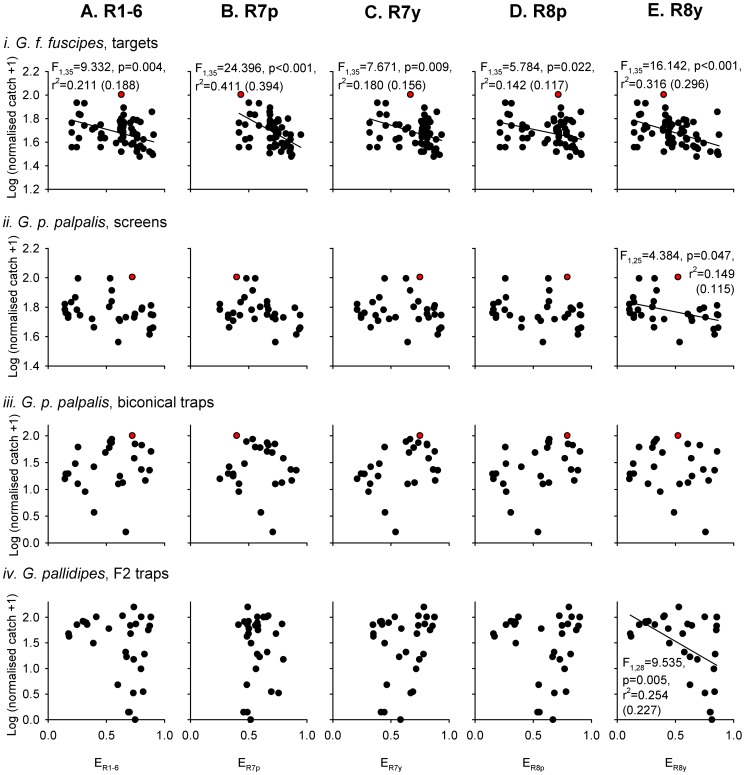
Female tsetse fly catches, and photoreceptor excitations, elicited by a range of visual baits. Female tsetse fly catches were obtained from three published field studies [5], [7], [11], as in Fig. 2, and have been log-transformed for analysis as in that figure. Data for the standard, phthalogen blue bait are indicated by the red data point in (i)–(iii). Tsetse catches are plotted against excitations calculated for each of the five main types of fly photoreceptor (A–E), based upon the reflectance spectra of visual baits used in the original studies. In each plot, data points are tsetse catches for every visual bait presentation in the original studies, but linear regression analyses were conducted on a reduced dataset in which each visual bait was represented once (against its mean normalised tsetse fly catch). The outcomes of statistical analyses are indicated where a significant relationship was identified (with adjusted r^2^ value in brackets). Sample sizes are as for Fig. 2.

Photoreceptor excitations were calculated from the reflectance spectra of 101 visual baits that were used in previous studies of tsetse fly capture in the field. These photoreceptor excitations are plotted against the log-transformed, normalised tsetse fly catch of each bait from the original studies (Figs. 2 and 3). Tsetse fly catch was significantly negatively related to calculated excitation in photoreceptor R8y in six out of the eight experimental datasets comprising different combinations of species, sex, and visual bait (linear regressions, p<0.05; Figs. 2 and 3). Of the other photoreceptor types, tsetse fly catch was significantly related to calculated excitations in a minority of the datasets (three each for R7p and R8p; two each for R7y and R1-6; Figs. 2 and 3). The responses of individual photoreceptor types provide achromatic (luminance) information, and receptors R1-6 are thought to be especially important in this role [31], [41]. Comparisons of the responses of different photoreceptor types, meanwhile, provide chromatic (spectral) information [41], [42], and previous studies have implicated this kind of information as an important determinant of tsetse fly attraction [5], [7], [11].

### Relative photoreceptor responses and R7y/R8y opponent interaction

**Figure 4:**
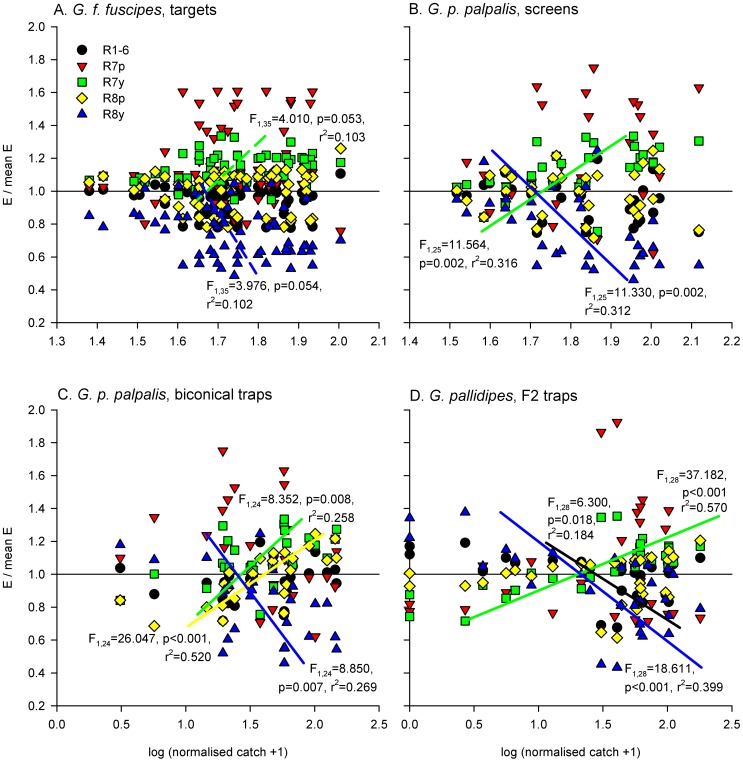
Male tsetse fly catches, and relative excitations of each photoreceptor type, elicited by visual baits. Here, calculated photoreceptor excitations for each visual bait are expressed as a proportion of the mean excitation across all five photoreceptor types to that same bait, and plotted against log-transformed normalised male tsetse fly catches across the four experiments (A–D). In each plot, the horizontal black line indicates mean excitation across all photoreceptor types. Log-transformed tsetse fly catches were regressed against normalised excitations for each photoreceptor type, and significant relationships are indicated. Note that the relationships indicated by dotted lines in panel A were not significant at p<0.05, but have been included for comparison across datasets. Other non-significant relationships have not been plotted. Sample sizes for data plots and statistical analyses as stated for Fig. 2.

**Figure 5:**
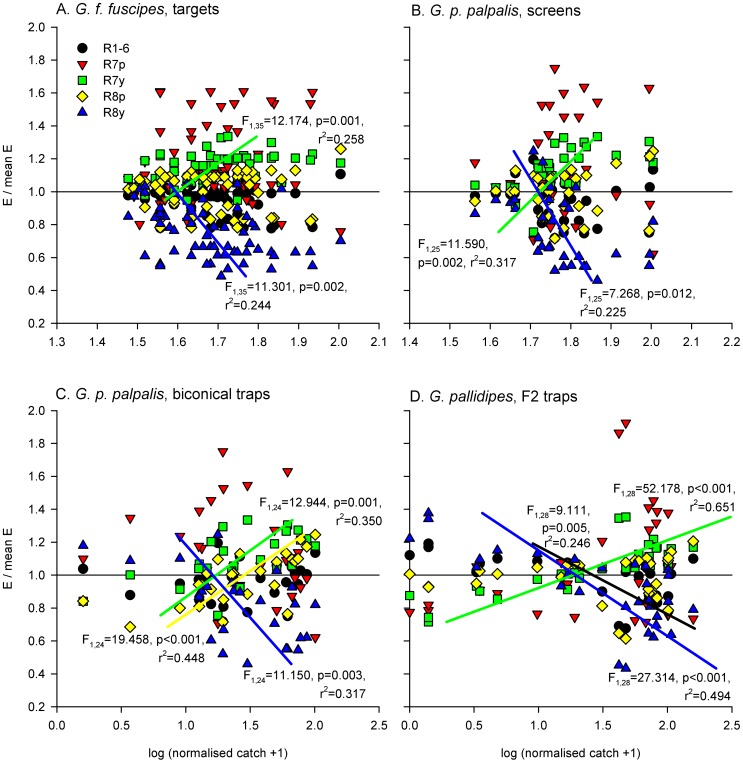
Female tsetse fly catches, and relative excitations of each photoreceptor type, elicited by visual baits. Relative photoreceptor excitations are calculated as in Fig. 4. In each plot, the horizontal black line indicates mean excitation across all photoreceptor types. Log-transformed tsetse fly catches were regressed against normalised excitations for each photoreceptor type, and significant relationships are indicated. Sample sizes for data plots and statistical analyses as stated for Fig. 2.

Relative photoreceptor excitations were visualised for each visual bait by expressing the excitation of each photoreceptor as a proportion of the mean excitation across all five photoreceptors (Figs. 4 and 5). Linear regression identified consistent, significant, positive relationships between the relative R7y photoreceptor response and log-transformed, normalised tsetse catch, and consistent, significant, negative relationships between the relative R8y receptor response and log-transformed, normalised tsetse catch (Figs. 4 and 5; note that these relationships were not significant at p<0.05 for male *G. f. fuscipes* but have been plotted for comparison; other non-significant relationships have not been plotted).

**Figure 6:**
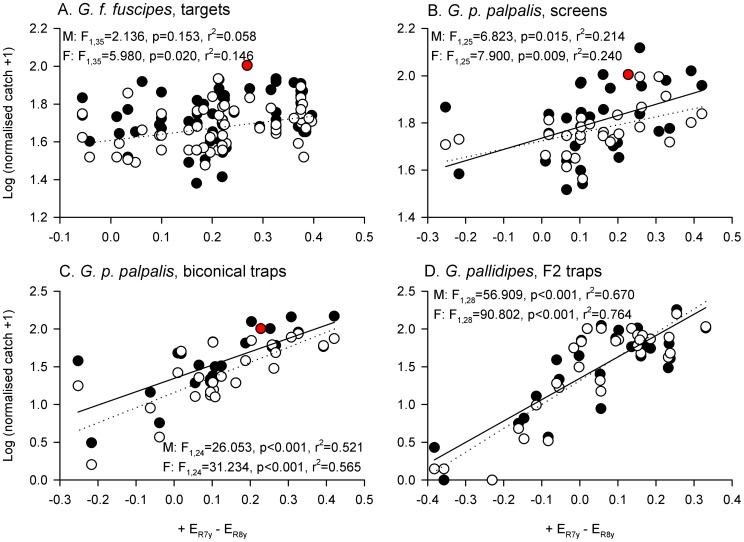
An opponent interaction between R7y and R8y is a good predictor of tsetse fly catches. The opponent interaction is calculated as + E_R7y_ – E_R8y_, and log-transformed, normalised male (filled circles) and female tsetse fly catches (open circles), are plotted against it. Fitted lines indicate significant relationships (p<0.05) for males (solid lines) and females (dotted lines), as determined by linear regression (see figure for details; note that the relationship for males in panel A was not significant). Red data points indicate phthalogen blue visual baits, which were not characterised by extreme values of this opponency index. Sample sizes for data plots and statistical analyses as stated in Fig. 2.

Since the ‘y’ form of the R7 and R8 receptor is the most abundant in the fly retina [17], and the relative excitations of these two cell types had consistent, opposite-sign relationships with tsetse fly catch, it was plausible that an opponent interaction between these two photoreceptor types could drive the attraction of tsetse flies to visual baits. An R7y/R8y opponent interaction is visualised in Fig. 6 as the difference in the calculated excitation of these two photoreceptors. Linear regression analysis identified a significant positive relationship between this opponency index and normalised tsetse fly catch for all datasets except that for male *G. f. fuscipes* (Fig. 6; note that linear regression analyses of these datasets using R7y and R8y excitations as separate predictors of tsetse fly catches are presented in the next section). Although this analysis implicates an R7y/R8y opponent interaction as an important mechanistic element underlying tsetse fly attraction to visual baits, this finding may not be particularly useful for the optimisation of visual baits for field use. This is because the tsetse fly catch of the preferred phthalogen blue target was not well predicted by this index – it was, for example, easily and consistently the most effective target in the study of Lindh et al. (2012) [5], but its calculated + R7y - R8y opponency index was not particularly unusual among the tested targets (red data points, Fig. 6).

Colour categorisation based on the signs of R7y/R8y and R7p/R8p opponent interactions has been proposed to explain colour discrimination learning in blowflies [43], so I next investigated whether this model of fly colour vision could better explain tsetse fly attraction to visual baits. However, blowfly-inspired colour categorisation had significant explanatory value for tsetse catches in only half of the datasets analysed, and was also unable to explain the unique effectiveness of phthalogen blue baits at catching tsetse flies (Figure S1; Table S2).

### Involvement of the other photoreceptors

Across the complete set of 101 visual baits from all three field studies, calculated excitations in the five classes of photoreceptor were significantly positively correlated with one another (table 1). Spearman's rank correlation coefficients were particularly high (>0.85) between photoreceptors R7y, R8p, and R1-6, indicating some redundancy in the stimulus information encoded by these photoreceptors. Correlations between the responses of these cells and those of R7p and R8y were weaker (with the exception of the correlation between R1-6 and R8y excitations), suggesting three more distinct sub-sets of visual information available from the photoreceptor array.

**Table 1:** Spearman's rank correlation coefficients between calculated photoreceptor excitations for 101 visual baits used in three published field studies [5], [7], [11].

	R1-6	R7p	R7y	R8p	R8y
**R1-6**					
**R7p**	0.645				
**R7y**	**0.890**	0.803			
**R8p**	**0.967**	0.659	**0.949**		
**R8y**	**0.938**	0.547	0.727	0.838	

All correlations significant at p<0.001; correlation coefficients>0.85 highlighted with bold text.

Due to the significant correlations between photoreceptor responses (table 1), I explored the possibility of a more complex interaction between photoreceptor types using multivariate partial least squares regression to generate models that predicted both the male and female tsetse catches within each of the four combinations of tsetse species and visual bait type (table 2). In these analyses, photoreceptors R7y and R8p were always positive contributors to the prediction of tsetse catches, whilst R8y and R7p were always negative contributors. R1-6 excitation was a negative predictor in three studies but a positive contributor in a fourth. Ranking the importance of these photoreceptor types by their standardised regression coefficients, and taking the median rank for each across the four studies, suggested that R7y and R8y were the most important predictors of tsetse catch (in line with the previously described analyses), followed by R7p and R8p, with R1-6 the least important predictors. The importance of R7p over R8p was supported by a number of additional lines of evidence: the R7p response was not strongly correlated with that of any other photoreceptor (table 1); standardised regression coefficients ranked R7p as a more important predictor than R8p in the target and screen datasets, in which tsetse catches provided a measure of attraction that could not have been confounded by varying landing or entering responses (see methods; table 2); and previous analyses indicated that UV wavelengths contribute negatively to tsetse fly attraction [5], [7], [11].

**Table 2:** Multivariate partial least squares regression analysis of log transformed tsetse fly catches using calculated photoreceptor excitations as predictors.

Species		*G. f. fuscipes*		*G. p. palpalis*		*G. p. palpalis*		*G. pallidipes*	
Bait		Targets		Screens		Biconical traps		F2 traps	
Sex		M.	F.	M.	F.	M.	F.	M.	F.
**R1-6**	**C.**	−0.006	−0.003	−0.112	−0.019	+0.186	+0.197	−1.063	−1.183
	***S.***	−*0.009^5^*	−*0.006^5^*	−*0.178^5^*	−*0.043^5^*	*+0.110^5^*	*+0.107^5^*	−*0.389^3^*	−*0.414^3^*
**R7p**	**C.**	−0.414	−0.594	−0.319	−0.361	−1.244	−1.404	−2.255	−1.797
	***S.***	−*0.421^1^*	−*0.700^1^*	−*0.397^3^*	−*0.664^3^*	−*0.587^4^*	−*0.611^4^*	−*0.381^4^*	−*0.291^5^*
**R7y**	**C.**	+0.166	+0.232	+0.404	+0.355	+1.485	+1.651	+4.240	+4.362
	***S.***	*+0.206^3^*	*+0.334^3^*	*+0.583^2^*	*+0.757^2^*	*+0.840^2^*	*+0.861^2^*	*+1.194^1^*	*+1.174^1^*
**R8p**	**C.**	+0.118	+0.171	+0.121	+0.187	+1.280	+1.421	+0.881	+0.852
	***S.***	*+0.186^4^*	*+0.312^4^*	*+0.197^4^*	*+0.450^4^*	*+0.798^3^*	*+0.818^3^*	*+0.329^5^*	*+0.304^4^*
**R8y**	**C.**	−0.238	−0.329	−0.457	−0.355	−1.808	−2.034	−2.636	−2.855
	***S.***	−*0.388^2^*	−*0.622^2^*	−*0.751^1^*	−*0.863^1^*	−*1.080^1^*	−*1.121^1^*	−*1.056^2^*	−*1.093^2^*
**constant**	**C.**	+1.956	+1.997	+1.951	+1.840	+1.456	+1.315	+1.795	+1.654
	***S.***	*0.000*	*0.000*	*0.000*	*0.000*	*0.000*	*0.000*	*0.000*	*0.000*
***LFs***		3		3		2		3*	
***r^2^***		0.207	0.541	0.422	0.435	0.794	0.836	0.817	0.858
***pred. r^2^***		0.015	0.381	0.174	0.194	0.727	0.771	0.754	0.809

Raw data from [5], [7], [11]. M. =  male; F. =  female; C. =  coefficient; S.  =  standardised coefficient; LFs  =  latent factors; pred. r^2^  =  predicted r^2^. The superscript following each standardised coefficient indicates its rank order of relative magnitude within each analysis.

* An initial model with 4 latent factors had a marginally improved fit, but the model selection plot indicated over-fitting. The parameters of this more complex model were: R1-6: −2.185 (−0.800), −2.437 (−0.852); R7p: −2.333 (−0.395), −1.884 (−0.305); R7y: +5.704 (+1.606), +5.997 (+1.614); R8p: −0.058 (−0.022), −0.199 (−0.071); R8y: −1.592 (−0.638), −1.688 (−0.646).

I next ran a series of linear regression models that introduced photoreceptor excitations sequentially in the order suggested by the above analysis (table 3). As expected, models containing R7y and R8y excitations provided a significant fit to the data in all but one case (male *G. f. fuscipes*), with adjusted r^2^ exceeding that for models containing only one photoreceptor (see Figs. 2 and 3), in six out of eight datasets. Addition of the R7p excitation parameter to these R7y/R8y models increased r^2^ and adjusted r^2^ for all datasets (table 3). A significant increase in r^2^ was achieved in six out of eight datasets, and of the remaining two, a significant fit to the data was obtained where previously there was none for male *G. f. fuscipes* (table 3). Subsequent addition of R8p and then R1-6 excitations to that model improved r^2^, but did so significantly in only one case for each of these additions (both trap datasets). Adjusted r^2^ was reduced by the addition of the R8p excitation parameter for two datasets, and by the addition of the R1-6 excitation parameter for four datasets (table 3). In four- and five-parameter regression models, variance inflation factors (VIFs) indicated high multicollinearity between R7y, R8p, and R1-6 (and in the five-parameter model, R8y) excitations, as was expected from their strong correlation (table 1). As a result, the signs and significance levels for the correlation coefficients were inconsistent between datasets. The observed collinearity supports the idea that R7y, R8p, and possibly R1-6 play essentially redundant roles in tsetse fly attraction, meaning that although it is possible that they contribute to the behaviour, their responses are not important in explaining the coarse detail of it for the purposes of visual bait optimisation. As implicated by statistical analyses reported so far, of several three-parameter regression models, that containing R7y excitation provided the best fit to tsetse fly catch data. However, R8p or R1-6 excitations could be substituted for those of R7y, and could still provide a significant explanation for the attraction data (although with reduced r^2^ in all but one case; Table S3).

**Table 3:** Linear regression analysis of log transformed tsetse fly catches with sequential addition of photoreceptor excitations as predictors.

			Model			
Study			R7y/R8y	+R7p	+R8p	+R1-6
***G.f. fuscipes***	**M, T**	*F test*	F_2,34_ = 2.450, p = 0.101	F_3,33_ = 2.923, **p = 0.048**	F_4,32_ = 2.151, p = 0.097	F_5,31_ = 1.736, p = 0.156
		*r^2^ (F test)*	0.126 (F_2,34_ = 2.450, p = 0.101)	0.210 (F_1,33_ = 3.508, p = 0.070)	0.212 (F_1,32_ = 0.081, p = 0.778)	0.219 (F_1,31_ = 0.269, p = 0.608)
		*Adj. r^2^*	0.075	0.138	0.113	0.093
***G. f. fuscipes***	**F, T**	*F test*	F_2,34_ = 7.859, **p = 0.002**	F_3,33_ = 13.669, **p<0.001**	F_4,32_ = 10.426, **p<0.001**	F_5,31_ = 8.081, **p<0.001**
		*r^2^ (F test)*	0.316 (F_2,34_ = 7.859, **p = 0.002**)	0.554 (F_1,33_ = 17.611, **p<0.001**)	0.566 (F_1,32_ = 0.865, p = 0.359)	0.566 (F_1,31_ = 0.003, p = 0.960)
		*Adj. r^2^*	0.276	0.514	0.512	0.496
***G. p. palpalis***	**M, S**	*F test*	F_2,24_ = 6.998, **p = 0.004**	F_3,23_ = 5.681, **p = 0.005**	F_4,22_ = 4.524, **p = 0.008**	F_5,21_ = 3.510, **p = 0.018**
		*r^2^ (F test)*	0.368 (F_2,24_ = 6.998, **p = 0.004**)	0.426 (F_1,23_ = 2.293, p = 0.144)	0.451 (F_1,22_ = 1.031, p = 0.321)	0.455 (F_1,21_ = 0.152, p = 0.700)
		*Adj. r^2^*	0.316	0.351	0.352	0.326
***G. p. palpalis***	**F, S**	*F test*	F_2,24_ = 4.393, **p = 0.024**	F_3,23_ = 6.867, **p = 0.002**	F_4,22_ = 6.767, **p = 0.001**	F_5,21_ = 5.671, **p = 0.002**
		*r^2^ (F test)*	0.268 (F_2,24_ = 4.393, **p = 0.024**)	0.473 (F_1,23_ = 8.918, **p = 0.007**)	0.552 (F_1,22_ = 3.883, p = 0.062)	0.575 (F_1,21_ = 1.130, p = 0.300)
		*Adj. r^2^*	0.207	0.404	0.470	0.473
***G. p. palpalis***	**M. B**	*F test*	F_2,23_ = 14.529, **p<0.001**	F_3,22_ = 21.572, **p<0.001**	F_4,21_ = 23.457, **p<0.001**	F_5,20_ = 18.195, **p<0.001**
		*r^2^ (F test)*	0.558 (F_2,23_ = 14.529, **p<0.001**)	0.746 (F_1,22_ = 16.312, **p = 0.001**)	0.817 (F_1,21_ = 8.132, **p = 0.010**)	0.820 (F_1,20_ = 0.295, p = 0.593)
		*Adj. r^2^*	0.520	0.712	0.782	0.775
***G. p. palpalis***	**F, B**	*F test*	F_2,23_ = 17.190, **p<0.001**	F_3,22_ = 35.270, **p<0.001**	F_4,21_ = 27.199, **p<0.001**	F_5,20_ = 23.017, **p<0.001**
		*r^2^ (F test)*	0.599 (F_2,23_ = 17.190, **p<0.001**)	0.828 (F_1,22_ = 29.232, **p<0.001**)	0.838 (F_1,21_ = 1.342, p = 0.260)	0.852 (F_1,20_ = 1.855, p = 0.188)
		*Adj. r^2^*	0.564	0.804	0.807	0.815
***G. pallidipes***	**M, F2**	*F test*	F_2,27_ = 33.203, **p<0.001**	F_3,26_ = 37.130, **p<0.001**	F_4,25_ = 28.569, **p<0.001**	F_5,24_ = 27.316, **p<0.001**
		*r^2^ (F test)*	0.711 (F_2,27_ = 33.203, **p<0.001**)	0.811 (F_1,26_ = 13.714, **p = 0.001**)	0.820 (F_1,25_ = 1.357, p = 0.255)	0.851 (F_1,24_ = 4.825, p = 0.038)
		*Adj. r^2^*	0.690	0.789	0.792	0.819
***G. pallidipes***	**F, F2**	*F test*	F_2,27_ = 51.533, **p<0.001**	F_3,26_ = 50.222, **p<0.001**	F_4,25_ = 41.703, **p<0.001**	F_5,24_ = 35.162, **p<0.001**
		*r^2^ (F test)*	0.792 (F_2,27_ = 51.533, **p<0.001**)	0.853 (F_1,26_ = 10.674, **p = 0.003**)	0.870 (F_1,25_ = 3.229, p = 0.084)	0.880 (F_1,24_ = 2.043, p = 0.166)
		*Adj. r^2^*	0.777	0.836	0.849	0.855

Raw data from [5], [7], [11]. Predictors were introduced sequentially, and for each model an F test of the fit of the regression is reported. Also reported for each model is r^2^, an F test of the change in r^2^ versus the previous model, and an r^2^ adjusted for the number of predictors in the model to allow comparison. Adjusted r^2^ values for models containing only one photoreceptor type as a predictor are provided in Figs. 2 and 3 for comparison. M. =  male; F. =  female; T =  target; S =  screen; B =  biconical trap; F2  =  F2 trap.

### Evaluating simple indices for the selection of visual baits prior to field testing

**Figure 7:**
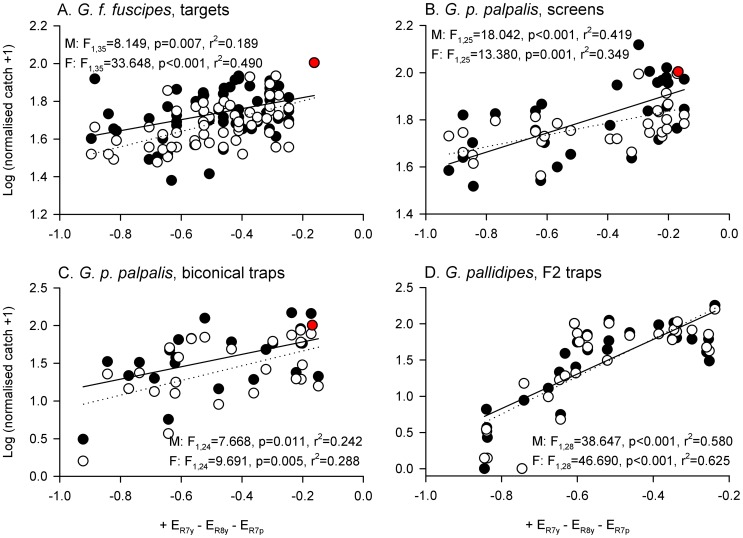
Opponent interaction between R7y, R8y, and R7p, can predict tsetse catches at visual baits. The opponent interaction is calculated as + E_R7y_ – E_R8y_ – E_R7p_, and log-transformed male (filled circles) and female tsetse fly catches (open circles), are plotted against it. Fitted lines indicate significant relationships (p<0.05) for males (solid lines) and females (dotted lines), as determined by linear regression (see figure for details). Red data points indicate phthalogen blue visual baits. Sample sizes for data plots and statistical analyses as stated in Fig. 2.

If candidate visual baits are to be assessed prior to field testing, it may be useful to represent the opponent interaction between photoreceptors implicated in the above analysis as a simply calculable index that ignores predictor weightings specific to individual studies, tsetse species, or visual baits. Such an index calculated by subtracting the unweighted excitations of R8y and R7p, from the unweighted excitation of R7y, is plotted in Fig. 7. Linear regression identified significant positive relationships between this simple opponency index and normalised tsetse fly catch for all eight sets of experimental data (Fig. 7; see also Table S4 for analysis of other simple indices calculated by linear combination of unweighted photoreceptor excitations). Although this simple index was a good predictor of tsetse catches at targets and screens, it was not the best-fitting index for trap catches which were, for example, better predicted by the + R7y - R8y opponency index considered above. However, in contrast to that index, phthalogen blue stimuli were characterised by more extreme values of the + R7y - R8y - R7p index, reflecting their observed effectiveness at catching tsetse flies in field experiments (red data points, Fig. 7).

## Discussion

I reanalysed data gathered in previous studies of tsetse fly catches at coloured visual baits using a model of fly photoreceptor excitations. This approach provides a biologically-authentic method of analysing the complete reflectance spectrum of a visual bait, as perceived by a fly. My reanalysis indicates that tsetse fly attraction to these visual baits can be explained by an opponent interaction to which a photoreceptor sensitive to blue wavelengths (R7y) makes a positive contribution, whilst photoreceptors sensitive to green-yellow (R8y) and UV wavelengths (R7p) make negative contributions. This finding is broadly consistent with the wavelengths of reflected light implicated in determining tsetse fly catches by the original studies [5], [7], [11], but provides more precision in mechanistic detail. The described approach will facilitate the appraisal of candidate visual baits prior to field testing, and can be used to analyse future studies of tsetse fly attraction.

At a coarse level, my findings are in agreement with those from the original investigations [5], [7], [11]. Monochromatic, luminance information from single photoreceptors did not provide a strong explanation for tsetse fly catches, which were better explained by the excitation of multiple photoreceptor types. Previous studies of *G. pallidipes*, *G. p. palpalis*, and *G. f. fuscipes*, found that tsetse fly catches at blue baits exceeded those at grey baits with a range of intensities [5], [7], [11], and regression models based upon catches of these species all implicated multiple reflectance bands in determining attraction [5], [7], [11]. In all three field studies, blue, UV, and green-yellow reflectance bands were implicated in determining attraction [5], [7], [11], corresponding to the peak sensitivities of three different photoreceptor classes and the findings of my reanalysis. Such comparison of the excitation of different classes of photoreceptor is plausible based upon neuroanatomical and behavioural studies of *Drosophila*. Photoreceptors R7 and R8 project to the medulla of the optic lobe, and neurons are found there that make appropriate contacts to facilitate comparison of outputs between and within ommatidia [44]. Furthermore, behavioural experiments reveal that *Drosophila* can discriminate colours based upon comparisons of excitation between different photoreceptor classes (including R1-6) [42].

Despite the overall agreement of my analysis with those of the original studies, there are differences in the detail. *Glossina pallidipes* catches were previously explained based upon visual bait reflectance in four colour bands: UV (300–410 nm), blue-green (410–520 nm), green-yellow-orange (520–615 nm), and red (615–700 nm) [11]. The eyes of higher flies have been thoroughly investigated (e.g. [17]), but no physiological study has yet identified a red-sensitive photoreceptor. Sensory information on reflectance in the red band is, therefore, unlikely to be available to the fly and was not represented in my analysis. A similar approach applied to *G. p. palpalis* conforms more closely to the findings of my reanalysis, with three wavelength bands explaining catches: UV (300–380 nm for biconical traps, 350–390 nm for screens), UV-blue (380–480 nm or 390–470 nm, respectively), and blue-green-yellow-red (480–620 nm, or 470–600 nm) [7]. The most visual-system-motivated study so far, on *G. f. fuscipes*, found 360 nm (UV) and 520 nm (green) to be repellent wavelengths, and 460 nm (blue) to be attractive [5]. The implication of that study is that the UV-blue receptor R7y (which has peak sensitivity at higher UV wavelengths) should have a repellent role, but my reanalysis (accounting for its shoulder of blue sensitivity), found that it was most likely to encode attractive stimulus information. The lower wavelength UV receptor R7p was implicated in a repellent role in my analysis, but point reflectance at its approximate peak sensitivity was not a significant predictor of attraction in the original study [5]. These discrepancies in fine detail indicate the importance of a more complete analysis that accounts for the entire sensitivity curve of a photoreceptor, rather than its peak sensitivity alone.

As in the original studies, my findings are based on the assumption that tsetse fly catches at coloured visual baits are indicators of attraction. However, two behavioural processes lead tsetse flies to become caught at visual baits: their attraction to approach the visual bait from a distance, and their decision to land upon it once close [10]. Target and screen experiments employed both surface and flanking nets to sample landing and circling flies, and are, therefore, presumed to be good indicators of attraction to approach the visual baits that are relatively unaffected by varying landing responses. These data were well-explained by the excitations of photoreceptors R7y, R8y, and R7p, and the simple opponency index based on these will be a useful tool for further optimisation of the small target visual baits with flanking nets that are currently advocated for controlling riverine tsetse flies [5], [8], [9]. Biconical and F2 trap experiments, meanwhile, only sampled tsetse flies that entered a trap, and their catches may have been affected by trap outer surface colours that stimulated or inhibited fly landing responses there in preference to trap entry [10], [11]. Addition of the R7p excitation parameter to a regression model containing R7y and R8y excitations did improve its fit to trap catch data, in line with the findings for targets and screens. However, PLS regressions suggested that R7p excitations were a relatively less important negative influence on tsetse catches at traps than they were at targets and screens (compare ranked standardised coefficients for this parameter in table 2), and the simple, unweighted + R7y – R8y – R7p opponency index fitted trap data less well than the index based on R7y and R8y excitations alone. Since high UV reflectance is reported to promote landing responses [10], [12], [13], it was expected to induce flies to land on the outer surface of a trap rather than entering it, enhancing the apparent negative effects of UV on attraction by further reducing the number of flies trapped. However, this seems not to have been the case. Since flies that land on a trap are subsequently more likely to enter it [14], it is plausible that enhanced landing responses as a result of UV reflectance ultimately led to enhanced trap catches, which then somewhat masked the negative effect of UV wavelengths on initial attraction in the trap datasets. Clearly the effects of visual bait colour on landing responses are an important topic for future investigation, and the methods described in this study can be applied to that problem. However, it will also be important to further investigate the behaviour of tsetse flies in the vicinity of traps (c.f. [14]), if these devices are to be optimised.

The methods applied in this study sought to identify the photoreceptor types that best explain tsetse fly attraction to visual baits based upon linear combination of their responses. However, the layout of opponent interactions between photoreceptors within the fly nervous system may differ from this simple scheme. R7y and R8y excitations appeared to be important predictors of tsetse attraction, and an opponent interaction between these receptors explained fly behaviour to a significant degree. The only studies of photoreceptor sensitivity in tsetse flies found an enhanced blue sensitivity in R7y and R8y due to the lack of a screening pigment that was attributed to dietary deficiency [18]. It is unknown whether wild tsetse experience this same elevated sensitivity to blue light, but such a pattern would still be consistent with these cells' involvement in attraction to visual baits, and might even enhance the attractiveness of blue surfaces. A negative input of R7p excitation to attraction was necessary to explain the particular attractiveness of phthalogen blue baits, and improved the fit to the tsetse catch data, but whether input from R7p interacts directly with that from R7y and R8y, is evaluated independently, or is processed in a separate opponent interaction with R8p (c.f. [43]), cannot be determined from the reported analyses. However, the latter scheme has intuitive appeal given the pairing of y- and p-type photoreceptors in separate ommatidia (although an R8p receptor has not yet been successfully recorded from in tsetse flies [18]). R8p, and especially R1-6, excitations did not appear to be as important as those of the other receptors in explaining the coarse detail of attraction. The highly-correlated nature of R7y, R8p, and R1-6 responses, and the fact that three-parameter regression models substituting R8p or R1-6 excitations for R7y excitations could still explain tsetse fly attraction reasonably well, help explain this apparent lack of importance in statistical analyses, and are consistent with recent findings that there is considerable redundancy among the opponent pathways in fly vision [42], [45].

Why tsetse flies are attracted towards blue stimuli remains an unanswered question. It has been suggested that, because shadow is illuminated only by skylight, which is bluer than direct illumination, flies might use blue as a chromatic cue to help them locate shaded resting places [46], [47]. Alternatively, since tsetse caught at visual baits are relatively starved [38] and, therefore, presumed to be host-seeking, It has been suggested that blue surfaces might contrast strongly with the surrounding vegetation, providing a strong signal of ‘not vegetation’ for the searching fly [36]. Natural spectra can be grouped within three broad categories: (i) living leaves, which have a green reflectance peak at 555 nm due to chlorophyll; (ii) most inorganic and organic surfaces, where reflectance increases gradually with wavelength; and (iii) diverse signalling colours like those of flowers and fruit which evolved so as to stand out from their background, but conform to no generalised template [48]. The innate attraction of many plant-feeding or plant-ovipositing insects towards green leaves can be explained by an opponent interaction in which a green-sensitive photoreceptor contributes positively, and a blue- (and/or UV-) sensitive photoreceptor contributes negatively [24], [49]. Turning this opponent interaction on its head (as demonstrated in this study for tsetse flies) would indeed provide a means to detect reflectance spectra that are not leaves. This may, perhaps, help explain why a variety of flower-feeding insects also display an innate blue preference, although in these cases the preference is altered by subsequent learning of flower colours [50], [51], [52]. It will be fascinating to probe these sensory ecological questions in future studies, and an understanding of attraction based upon photoreceptor excitations will provide a basis with which to do so.

The opponent model presented in this study refines previous analyses of tsetse fly catches at coloured visual baits, and will facilitate the kind of detailed spectral analysis of candidate baits called for by Lindh et al. (2012) [5]. To promote such analyses, a tool for the calculation of photoreceptor excitations is available as ESM, and this study has demonstrated that an easily calculable opponency index between the photoreceptor excitations with greatest explanatory power has good predictive value for tsetse catches, especially at small targets and screens. Thus, baits might be evaluated in this way from their reflectance spectra alone, prior to confirmatory field testing. Future studies may fine tune the model developed in this paper using more specific quantifications of background reflectance and illumination, and this approach is likely to explain the variations in the relative attractiveness of colours between habitats that have been noted in other tsetse species [38]. Improvements to visual baits in other respects such as shape [53], odour cues [54], and placement [6], will all contribute to the further optimisation of these vitally important public health devices for sub-Saharan Africa.

## Supporting Information

Dataset S1 **Collated dataset.** The dataset used in statistical analysis, including calculated photoreceptor excitations for each visual bait.
(XLSX)

Figure S1 **Tsetse fly catches and visual bait colour categorisation according to the model of Troje (1993).** On the basis of conditioned colour discrimination experiments in *Lucilia* spp., Troje (1993) [43] proposed that blowflies perceive four colour categories determined by the sign of the output from each of two opponent interactions: R7y – R8y (‘y’), and R7p – R8p (‘p’). The proposed colour categories are thus: y+p-, y+p+, y-p-, and y-p+. Although such a scheme provided a good explanation for learned discriminations in *Lucilia*, this was not the case for attraction to visual baits in tsetse flies. Overall, these colour categories significantly explained normalised tsetse fly catches in four out of eight datasets (Table S2). However, the majority of visual baits were assigned to categories y+p- (including the phthalogen blue baits), and y+p+ (Ns above bars indicate the number of baits assigned to each category); colour category was a significant predictor of differences in normalised tsetse fly catch between these two colour categories in only two of the datasets (Table S2). Furthermore, the large number of baits in the y+p- category meant that the unique attractiveness of the phthalogen blue target was not explained. Error bars  =  standard deviation. Data from [5], [7], [11], with each visual bait represented once.
(TIF)

Table S1 **Fly photoreceptor excitation calculator.** A spreadsheet that calculates photoreceptor excitations, and simple opponency indices, from visual bait reflectance spectra.
(XLSX)

Table S2 **Linear regression analysis of tsetse fly catches using dummy-coded blowfly colour categories as predictors.** Data from [5], [7], [11]. Three of the four colour categories (see Figure S1) were 1-0 dummy coded, with category y+p- (that of the phthalogen blue standards) as the reference. Colour categories significantly predicted tsetse fly catches in four out of eight datasets (Regression). However, the y+p+ category only predicted a significant decrease in tsetse fly catch versus the y+p- category in two of the datasets, whilst the majority of visual baits were categorised y+p-, or y+p+ (see Figure S1). M =  male, F =  female, T =  target, S =  screen, B =  biconical trap, F2  =  F2 trap.
(DOCX)

Table S3 **Linear regression analysis of tsetse fly catches using excitations of three photoreceptor types as predictors.** Data from [5], [7], [11], For each dataset, linear regression was conducted using R8y, R7p, and one other photoreceptor (R7y, R8p, or R1-6) as predictors. An F test of the significance of each regression model is reported (reg.), below which are the unstandardised regression coefficients for each predictor (coeff.) and the constant (con.). Asterisks indicate significant differences from zero (t-tests; *<0.05, **<0.01). Adjusted r^2^ values indicate the overall fit of each regression model. M. =  male; F. =  female; T =  target; S =  screen; B =  biconical trap; F2  =  F2 trap.
(DOCX)

Table S4 **Analysis of tsetse fly catches using simple indices calculated from unweighted photoreceptor excitations as predictors.** Data from [5], [7], [11], Opponency indices were calculated by the addition or subtraction of photoreceptor excitations as indicated in the column headings. For each dataset, an F test for the fit of a regression with each opponency index as a predictor is reported (Reg.), with the r^2^ for that regression. M. =  male; F. =  female; T =  target; S =  screen; B =  biconical trap; F2  =  F2 trap.
(DOCX)
