# Correction: A Colour Opponent Model That Explains Tsetse Fly Attraction to Visual Baits and Can Be Used to Investigate More Efficacious Bait Materials

**DOI:** 10.1371/journal.pntd.0003723

**Published:** 2015-04-17

**Authors:** Roger D. Santer

There is an error in the second paragraph under the heading “Tsetse fly data”. The size of the cloth targets used is incorrect. The correct size is: 0.0625 m^2^.
