# Supplementary figures and images for: A Colour Opponent Model That Explains Tsetse Fly Attraction to Visual Baits and Can Be Used to Investigate More Efficacious Bait Materials
Source: PLoS Negl Trop Dis. 2014 Dec 4;8(12):e3360. doi: 10.1371/journal.pntd.0003360 (PMC4256293; doi:10.1371/journal.pntd.0003360)

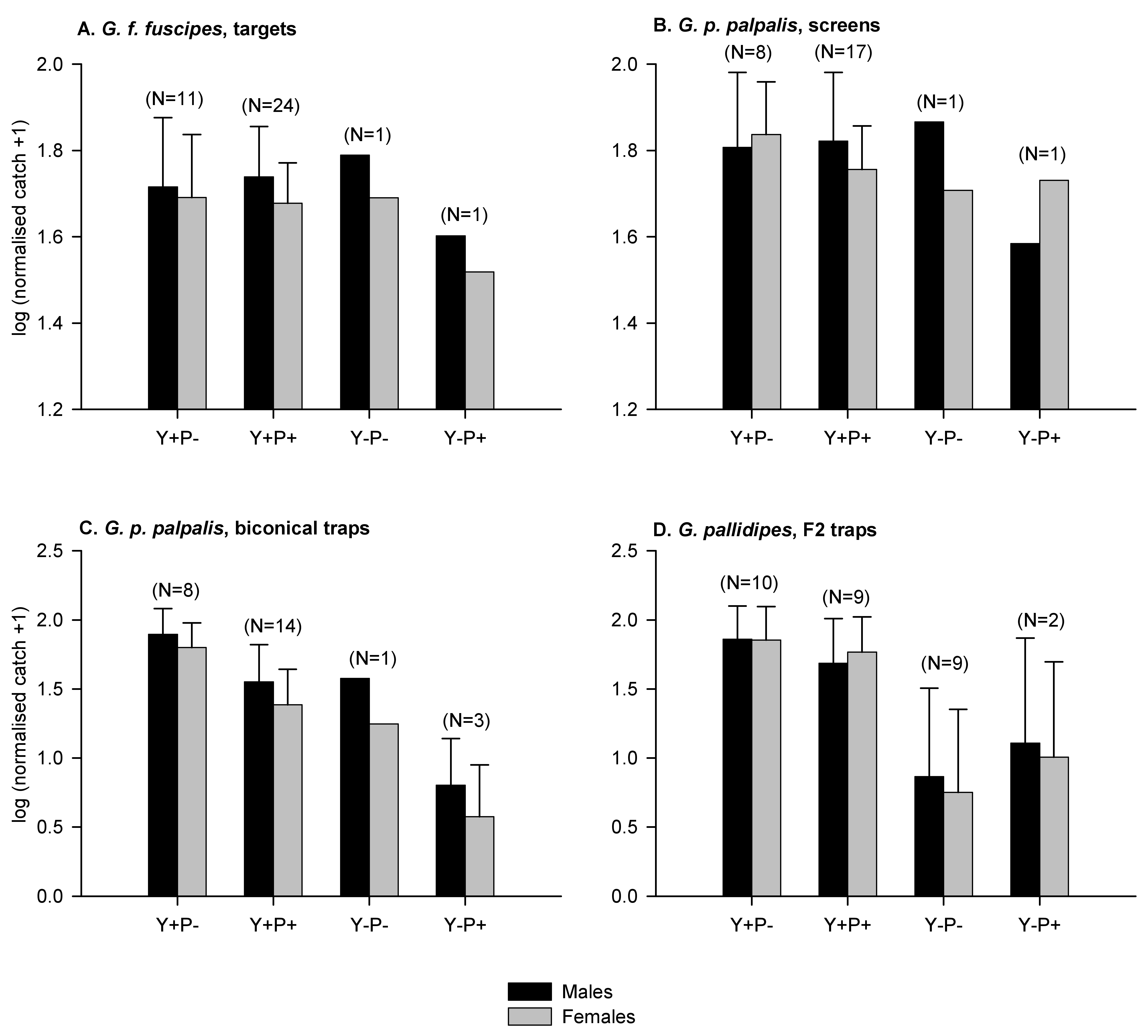

Supplement: Figure S1 — Tsetse fly catches and visual bait colour categorisation according to the model of Troje (1993). On the basis of conditioned colour discrimination experiments in Lucilia spp., Troje (1993) [43] proposed that blowflies perceive four colour categories determined by the sign of the output from each of two opponent interactions: R7y – R8y (‘y’), and R7p – R8p (‘p’). The proposed colour categories are thus: y+p-, y+p+, y-p-, and y-p+. Although such a scheme provided a good explanation for learned discriminations in Lucilia, this was not the case for attraction to visual baits in tsetse flies. Overall, these colour categories significantly explained normalised tsetse fly catches in four out of eight datasets (Table S2). However, the majority of visual baits were assigned to categories y+p- (including the phthalogen blue baits), and y+p+ (Ns above bars indicate the number of baits assigned to each category); colour category was a significant predictor of differences in normalised tsetse fly catch between these two colour categories in only two of the datasets (Table S2). Furthermore, the large number of baits in the y+p- category meant that the unique attractiveness of the phthalogen blue target was not explained. Error bars = standard deviation. Data from [5], [7], [11], with each visual bait represented once. (TIF) [file pntd.0003360.s002.tif]
