# Supplementary material for: A Colour Opponent Model That Explains Tsetse Fly Attraction to Visual Baits and Can Be Used to Investigate More Efficacious Bait Materials
Source: PLoS Negl Trop Dis. 2014 Dec 4;8(12):e3360. doi: 10.1371/journal.pntd.0003360 (PMC4256293; doi:10.1371/journal.pntd.0003360)
Supplement: Table S2 — Linear regression analysis of tsetse fly catches using dummy-coded blowfly colour categories as predictors. Data from [5], [7], [11]. Three of the four colour categories (see Figure S1) were 1-0 dummy coded, with category y+p- (that of the phthalogen blue standards) as the reference. Colour categories significantly predicted tsetse fly catches in four out of eight datasets (Regression). However, the y+p+ category only predicted a significant decrease in tsetse fly catch versus the y+p- category in two of the datasets, whilst the majority of visual baits were categorised y+p-, or y+p+ (see Figure S1). M = male, F = female, T = target, S = screen, B = biconical trap, F2 = F2 trap. (DOCX) [file pntd.0003360.s004.docx]

| **Species** | **Details** | **Regression** | **Dummy variables (reference = y+p-)** | | |
| --- | --- | --- | --- | --- | --- |
|  |  |  | **y+p+** | **y-p+** | **y-p-** |
| ***G. f. fuscipes*** | M./T. | F_3,33_=0.461, p=0.712  (*r^2^= 0.040)* | t=0.482, p=0.633 | t=-0.826, p=0.414 | t=0.533, p=0.597 |
|  | F./T. | F_3,33_=0.728, p=0.543  (*r^2^= 0.062)* | t=-0.338, p=0.737 | t=-1.474, p=0.150 | t=-0.009, p=0.993 |
| ***G. p. palpalis*** | M./S. | F_3,23_=0.704, p=0.559  (*r^2^= 0.084)* | t=0.215, p=0.832 | t=-1.282, p=0.213 | t=0.341, p=0.736 |
|  | F./S. | F_3,23_=1.252, p=0.314  (*r^2^= 0.140)* | t=-1.759, p=0.092 | t=-0.935, p=0.360 | t=-1.138, p=0.267 |
| ***G. p. palpalis*** | M./B. | F_3,22_=13.739, **p<0.001**  (*r^2^= 0.652)* | t=-3.072, **p=0.006** | t=-6.405, **p<0.001** | t=-1.195, p=0.245 |
|  | F./B. | F_3,22_=17.807, **p<0.001**  (*r^2^= 0.708)* | t=-3.741, **p=0.001** | t=-7.233, **p<0.001** | t=-2.083, **p=0.049** |
| ***G. pallidipes*** | M./F2 | F_3,26_=9.176, **p<0.001**  (*r^2^= 0.514)* | t=-0.851, p=0.403 | t=-2.178, **p=0.039** | t=-4.848, **p<0.001** |
|  | F./F2 | F_3,26_=14.427, **p<0.001**  (*r^2^= 0.625)* | t=-0.456, p=0.652 | t=-2.661, **p=0.013** | t=-5.833, **p<0.001** |
