# Supplementary material for: A Colour Opponent Model That Explains Tsetse Fly Attraction to Visual Baits and Can Be Used to Investigate More Efficacious Bait Materials
Source: PLoS Negl Trop Dis. 2014 Dec 4;8(12):e3360. doi: 10.1371/journal.pntd.0003360 (PMC4256293; doi:10.1371/journal.pntd.0003360)
Supplement: Table S3 — Linear regression analysis of tsetse fly catches using excitations of three photoreceptor types as predictors. Data from [5], [7], [11], For each dataset, linear regression was conducted using R8y, R7p, and one other photoreceptor (R7y, R8p, or R1-6) as predictors. An F test of the significance of each regression model is reported (reg.), below which are the unstandardised regression coefficients for each predictor (coeff.) and the constant (con.). Asterisks indicate significant differences from zero (t-tests; *<0.05, **<0.01). Adjusted r2 values indicate the overall fit of each regression model. M. = male; F. = female; T = target; S = screen; B = biconical trap; F2 = F2 trap. (DOCX) [file pntd.0003360.s005.docx]

| **Species** |  | **Model** | | |
| --- | --- | --- | --- | --- |
| Details |  | **R7y/R8y/R7p** | **R8p/R8y/R7p** | **R1-6/R8y/R7p** |
| ***G. f. fuscipes*** | *Reg.* | F_3,33_=2.923, p=0.048 | F_3,33_=2.784, p=0.056 | F_3,33_=2.741, p=0.059 |
| M./T. | *Coeff.* | +0.333/-0.207/-0.502 | +0.216/-0.252/-0.343 | +0.308/-0.335/-0.354 |
|  | *Con.* | +1.957** | +1.964** | +1.964** |
|  | *Adj r^2^* | 0.138 | 0.129 | 0.127 |
| ***G. f. fuscipes*** | *Reg.* | F_3,33_=13.669, p<0.001 | F_3,33_=12.201, p<0.001 | F_3,33_=12.167, p<0.001 |
| F./T. | *Coeff.* | +0.485**/-0.283**/-0.729** | +0.304*/-0.342**/-0.495** | +0.451*/-0.472**/-0.514** |
|  | *Con.* | +1.997** | +2.009** | +2.008** |
|  | *Adj r^2^* | 0.514 | 0.483 | 0.482 |
| ***G. p. palpalis*** | *Reg.* | F_3,23_=5.681, p=0.005 | F_3,23_=4.920, p=0.009 | F_3,23_=4.726, p=0.010 |
| M./S. | *Coeff.* | +0.494/-0.466*/-0.396 | +0.354/-0.567*/-0.153 | +0.514/-0.734*/-0.146 |
|  | *Con.* | +1.955** | +1.956** | +1.950** |
|  | *Adj r^2^* | 0.351 | 0.311 | 0.301 |
| ***G. p. palpalis*** | *Reg.* | F_3,23_=6.867, p=0.002 | F_3,23_=4.407, p=0.014 | F_3,23_=4.447, p=0.013 |
| F./S. | *Coeff.* | +0.589**/-0.282*/-0.507** | +0.423*/-0.403*/-0.217 | +0.668*/-0.647**/-0.217 |
|  | *Con.* | +1.848** | +1.849** | +1.838** |
|  | *Adj r^2^* | 0.404 | 0.282 | 0.285 |
| ***G. p. palpalis*** | *Reg.* | F_3,22_=21.572, p<0.001 | F_3,22_=32.535, p<0.001 | F_3,22_=28.102, p<0.001 |
| M./B. | *Coeff.* | +2.961**/-1.296**/-1.769** | +2.663**/-2.283**/-0.410 | +4.139**/-3.762**/-0.412 |
|  | *Con.* | +1.456** | +1.406** | +1.344** |
|  | *Adj r^2^* | 0.712 | 0.791 | 0.765 |
| ***G. p. palpalis*** | *Reg.* | F_3,22_=35.270, p<0.001 | F_3,22_=33.194, p<0.001 | F_3,22_=25.096, p<0.001 |
| F./B. | *Coeff.* | +3.387**/-1.457**/-2.115** | +2.891**/-2.481**/-0.511 | +4.427**/-4.033**/-0.500 |
|  | *Con.* | +1.323** | +1.273** | +1.209** |
|  | *Adj.r^2^* | 0.804 | 0.794 | 0.743 |
| ***G. pallidipes*** | *Reg.* | F_3,26_=37.130, p<0.001 | F_3,26_=16.216, p<0.001 | F_3,26_=9.167, p<0.001 |
| M./F2 | *Coeff.* | +4.548**/-2.955**/-2.385** | +3.856**/-3.952**/-1.419 | +5.377**/-5.563**/-1.227 |
|  | *Con.* | +1.750** | +2.209** | +2.076** |
|  | *Adj r^2^* | 0.789 | 0.612 | 0.458 |
| ***G. pallidipes*** | *Reg.* | F_3,26_=50.222, p<0.001 | F_3,26_=18.953, p<0.001 | F_3,26_=11.536, p<0.001 |
| F./F2 | *Coeff.* | +4.599**/-3.262**/-1.941** | +3.813**/-4.209**/-0.915 | +5.392**/-5.863**/-0.754 |
|  | *Con.* | +1.620** | +2.072** | +1.947** |
|  | *Adj r^2^* | 0.836 | 0.650 | 0.522 |
