# Supplementary material for: A Colour Opponent Model That Explains Tsetse Fly Attraction to Visual Baits and Can Be Used to Investigate More Efficacious Bait Materials
Source: PLoS Negl Trop Dis. 2014 Dec 4;8(12):e3360. doi: 10.1371/journal.pntd.0003360 (PMC4256293; doi:10.1371/journal.pntd.0003360)
Supplement: Table S4 — Analysis of tsetse fly catches using simple indices calculated from unweighted photoreceptor excitations as predictors. Data from [5], [7], [11], Opponency indices were calculated by the addition or subtraction of photoreceptor excitations as indicated in the column headings. For each dataset, an F test for the fit of a regression with each opponency index as a predictor is reported (Reg.), with the r2 for that regression. M. = male; F. = female; T = target; S = screen; B = biconical trap; F2 = F2 trap. (DOCX) [file pntd.0003360.s006.docx]

|  |  |  | **Simple opponency index** | | | |
| --- | --- | --- | --- | --- | --- | --- |
| **Species** | Details |  | **+R7y-R8y** | **+R7y-R8y**  **-R7p** | **+R7y-R8y**  **+R8p-R7p** | **+R7y-R8y**  **+R8p-R7p**  **-R1-6** |
| ***G. f. fuscipes*** | M./T. | *Reg.* | F_1,35_=2.136, p=0.153 | F_1,35_=8.149, **p=0.007** | F_1,35_=1.350, p=0.253 | F_1,35_=7.908, **p=0.008** |
|  |  | *r^2^* | 0.058 | 0.189 | 0.037 | 0.184 |
| ***G. f. fuscipes*** | F./T. | *Reg.* | F_1,35_=5.980, **p=0.020** | F_1,35_=33.648, **p<0.001** | F_1,35_=3.989, p=0.054 | F_1,35_=31.474, **p<0.001** |
|  |  | *r^2^* | 0.146 | 0.490 | 0.102 | 0.473 |
| ***G. p. palpalis*** | M./S. | *Reg.* | F_1,25_=6.823, **p=0.015** | F_1,25_=18.042, **p<0.001** | F_1,25_=2.198, p=0.151 | F_1,25_=18.048, **p<0.001** |
|  |  | *r^2^* | 0.214 | 0.419 | 0.081 | 0.419 |
| ***G. p. palpalis*** | F./S. | *Reg.* | F_1,25_=7.900, **p=0.009** | F_1,25_=13.380, **p=0.001** | F_1,25_=8.576, **p=0.007** | F_1,25_=14.699, **p=0.001** |
|  |  | *r^2^* | 0.240 | 0.349 | 0.255 | 0.370 |
| ***G. p. palpalis*** | M./B. | *Reg.* | F_1,24_=26.053, **p<0.001** | F_1,24_=7.668, **p=0.011** | F_1,24_=79.882, **p<0.001** | F_1,24_=13.765, **p=0.001** |
|  |  | *r^2^* | 0.521 | 0.242 | 0.769 | 0.364 |
| ***G. p. palpalis*** | F./B. | *Reg.* | F_1,24_=31.234, **p<0.001** | F_1,24_=9.691, **p=0.005** | F_1,24_=100.503, **p<0.001** | F_1,24_=17.228, **p<0.001** |
|  |  | *r^2^* | 0.565 | 0.288 | 0.807 | 0.418 |
| ***G. pallidipes*** | M./F2 | *Reg.* | F_1,28_=56.909, **p<0.001** | F_1,28_=38.647, **p<0.001** | F_1,28_=22.751, **p<0.001** | F_1,28_=62.127, **p<0.001** |
|  |  | *r^2^* | 0.670 | 0.580 | 0.448 | 0.689 |
| ***G. pallidipes*** | F./F2 | *Reg.* | F_1,28_=90.802, **p<0.001** | F_1,28_=46.690, **p<0.001** | F_1,28_=19.134, **p<0.001** | F_1,28_=75.723, **p<0.001** |
|  |  | *r^2^* | 0.764 | 0.625 | 0.406 | 0.730 |
